# A note on the impact of late diagnosis on HIV/AIDS dynamics: a mathematical modelling approach

**DOI:** 10.1186/s13104-020-05179-y

**Published:** 2020-07-16

**Authors:** J. Mushanyu

**Affiliations:** Department of Mathematics, University of Zimbabwe, Mount Pleasant, Box MP 167, Harare, Zimbabwe

**Keywords:** HIV, ART treatment, Basic reproduction number, Stability analysis, Numerical simulations

## Abstract

**Objectives:**

The global incidence of HIV infection is not significantly decreasing, especially in sub-Saharan African countries. Though there is availability and accessibility of free HIV services, people are not being diagnosed early for HIV, and hence HIV-related mortality remains significantly high. We formulate a mathematical model for the spread of HIV using non linear ordinary differential equations in order to investigate the impact of late diagnosis of HIV on the spread of HIV.

**Results:**

The results suggest the need to encourage early initiation into HIV treatment as well as promoting HIV self-testing programs that enable more undiagnosed people to know their HIV status in order to curtail the continued spread of HIV.

## Introduction

Antiretroviral therapy (ART) has successfully transformed human immunodeficiency virus (HIV) infection from a fatal to a manageable chronic disease [1]. Nonetheless, there remains critical factors to be addressed along with the roll out of effective ART regimens in order to eradicate HIV. We seek to investigate the impact of late diagnosis on the transmission dynamics of HIV. Mathematical modeling of HIV dynamics is quite advanced, see for instance the following works on HIV and the references therein [2–9].

We extend a more recent HIV/AIDS mathematical model developed by Omondi et al. [8] to investigate the impact of late diagnosis on the spread and control of HIV. In their work, Omondi et al. [8] proposed a five state deterministic compartmental model for the time evolution of population states to study the trend of HIV infection in Kenya. The model was premised on dividing the infected classes according to CD4^+^ T cell counts in the blood. For more information about the description of parameters and model analysis, readers are referred to Omondi et al. [8].

The paper is arranged as follows; in "Main text" section, we formulate and establish the basic properties of the model. The model is analysed for stability in this section. In "Results and discussion" section, we carry out some numerical simulations. Parameter estimation and numerical results are also presented in this section. The paper is concluded in "Conclusions" section.

## Main text

### The model

We propose a five state compartmental model for HIV that takes into account untimely initiation of HIV positive individuals into ART. The human population comprises classes; *S*(*t*), $I_1(t)$, $I_2(t)$, $I_{A1}(t)$ and $I_{A2}(t)$. The class *S*(*t*) represents the population at high risk of HIV infection. Upon acquiring HIV infection, susceptible individuals move to infection class which is divided into two stages according to CD4^+^ T cell count in the blood. The infectives class $I_1$ comprise of individuals with CD4^+^ T cell count $\ge 350$/μL. Individuals in class $I_1$ are assumed to be having a lower viral load and hence are considered to be the new infections. Individuals in class $I_1$ progress to the second stage of infection $I_2$ at a rate given by $\delta$. This class consists of individuals with CD4^+^ T cell count in the range $200-350$/μL. Individuals in this stage are assumed to be having high viral load. Individuals in class $I_1$ are initiated into ART treatment at a rate given by $\sigma _1$. In this paper, we develop a mathematical model that takes into account the effect of late initiation into ART treatment of HIV positive patients. We define initiation of HIV positive individuals in stage $I_2$ into ART treatment by the expression

1 $$\begin{aligned} H\left( I_2\right) =\frac{\sigma _2 I_2}{1+rI_2}. \end{aligned}$$

Here, $\sigma _2$ represent the maximum treatment uptake per unit of time for individuals in class $I_2$ and *r* measures the extent of the effect of late initiation into ART treatment. Firstly, observe that for small $I_2$, $H(I_2)\approx \sigma _2I_2$. Secondly, observe that for large $I_2$, $H(I_2)\approx \sigma _2/r$. Finally, when $r=0$, we obtain $H(I_2)= \sigma _2I_2$, which is the case considered in Omondi et al. [8]. Individuals in class $I_{A1}$ move to the class $I_{A2}$ through a deteriorative process at a rate given by $\gamma _1$ whereas individuals in class $I_{A2}$ move to the class $I_{A1}$ through an ameliorative process at a rate given by $\gamma _2$. In this model, we exclude the class of full blown AIDS patients as these are usually hospitalised and/or sexually inactive and hence their contribution to new HIV infections is negligible [8]. The total human population is thus given by

$$\begin{aligned} N(t)=S(t)+I_1(t)+I_2(t)+I_{A1}(t)+I_{A2}(t). \end{aligned}$$

Susceptible humans are recruited into the system through births or immigration at a constant rate $\Lambda$. Susceptible individuals acquire new HIV infections at a rate given by

2 $$\begin{aligned} \lambda =\frac{\beta _1I_1+\beta _2I_2+\beta _3I_{A1}+\beta _4I_{A2}}{N} \end{aligned}$$

where $\beta _1$, $\beta _2$, $\beta _3$ and $\beta _4$ denote the HIV transmission rates between susceptible individuals and infectious individuals. We assume that individuals in each compartment are indistinguishable and there is homogeneous mixing. Individuals in classes $I_2$ and $I_{A2}$ experience disease related death at rates given respectively by $\omega _1$ and $\omega _2$. The natural death rate of the general population is represented by $\mu$. The differential equations for the model are given as follows;

3 $$\begin{aligned} \left\{ \begin{array}{l} \dfrac{dS}{dt}=\Lambda -\lambda S-\mu S, \\ \dfrac{dI_1}{dt}=\lambda S -(\mu +\delta +\sigma _1)I_1,\\ \dfrac{dI_2}{dt}=\delta I_1-(\mu +\omega _1)I_2-H(I_2),\\ \dfrac{dI_{A1}}{dt}=\sigma _1 I_1-(\mu +\gamma _1)I_{A1}+\gamma _2I_{A2},\\ \dfrac{dI_{A2}}{dt}=H(I_2)-(\mu +\gamma _2 +\omega _2)I_{A2}+\gamma _1 I_{A1}, \end{array} \right. \end{aligned}$$

with the initial conditions:

$$\begin{aligned} S(0)=S_{0}>0,\,I_1(0)=I_{10}\ge 0,\,I_2(0)=I_{20}\ge 0,\,I_{A1}(0)=I_{A10}\ge 0,\,I_{A2}(0)=I_{A20}\ge 0, \end{aligned}$$

where we assume that all the model parameters are positive.

### Analysis of the model

#### Positivity of solutions

The following theorem (Theorem 1) entails that all the state variables remain non-negative and the solutions of system (3) with positive initial conditions will remain positive for all $t > 0$.

##### Theorem 1

*Given that the initial conditions of system* (3) *are*$S(0)>0$, $I_1(0)>0$, $I_2(0)>0$, $I_{A1}(0)>0$*and*$I_{A2}(0)>0$. *There exists*$(S(t),I_1(t),I_2(t),I_{A1}(t),I_{A2}(t)): (0,\infty )\rightarrow (0,\infty )$*which solve system* (3).

For more details on the proof of Theorem 1, we refer the reader to [8].

#### Invariant region

The feasible region for system (3) is given by

4 $$\begin{aligned} \Omega =\left\{ (S,I_1,I_2,I_{A1},I_{A2})\in {\mathbb {R}}^{5}_{+}\,|\,N\le \frac{\Lambda }{\mu }\right\} . \end{aligned}$$

Results to verify that the region $\Omega$ is positively invariant with respect to system (3) can be obtained as given in [8].

#### Disease-free equilibrium and the basic reproduction number

The model has a disease-free equilibrium given by

$$\begin{aligned} {\mathcal {D}}^f=\left( S^f,I^f_1,I^f_2,I^f_{A_1},I^f_{A_2}\right) =\left( \frac{\Lambda }{\mu },0,0,0,0\right) , \end{aligned}$$

a scenario depicting a disease-free state in the community or society. The basic reproduction number ${\mathcal {R}}_0$ of the model, is defined herein as the average number of people infected by each HIV infected individual during his/her infectious period in a population of completely susceptible individuals. The determination of ${\mathcal {R}}_0$ is done using the next generation matrix approach [10]. It works out that, the basic reproduction number of system (3) is given by:

5 $$\begin{aligned} \left\{ \begin{array}{l} {\mathcal {R}}_0={\mathcal {R}}_{I_1}+{\mathcal {R}}_{I_2}+{\mathcal {R}}_{I_{A1}}+{\mathcal {R}}_{I_{A2}}\,\,\,\text{ where }\,\,\,\\ {\mathcal {R}}_{I_1}=\dfrac{\beta _1}{h_1},\quad {\mathcal {R}}_{I_2}=\dfrac{\beta _2 \delta }{h_1 h_2},\quad {\mathcal {R}}_{I_{A1}}=\dfrac{\beta _3 \left( \gamma _2 \delta \sigma _2+h_2 h_4 \sigma _1\right) }{h_1 h_2h_3h_4 \left( 1-\Phi \right) }\,\,\text{ and }\\ {\mathcal {R}}_{I_{A2}}=\dfrac{\beta _4 \left( \gamma _1 h_2 \sigma _1+\delta h_3 \sigma _2\right) }{h_1 h_2h_3h_4 \left( 1-\Phi \right) }\quad \text{ with }\quad \Phi =\dfrac{\gamma _1\gamma _2}{h_3h_4}, \quad h_1=\mu +\delta +\sigma _1,\\ h_2=\mu +\sigma _2+\omega _1,\quad h_3=\mu +\gamma _1 \quad \text{ and } \quad h_4=\mu +\gamma _2+\omega _2. \end{array} \right. \end{aligned}$$

Here, the four sub-reproduction numbers ${\mathcal {R}}_{I_1}$, ${\mathcal {R}}_{I_2}$, ${\mathcal {R}}_{I_{A_1}}$ and ${\mathcal {R}}_{I_{A_2}}$ represent the contributions of individuals in compartments $I_1$, $I_2$, $I_{A_1}$ and $I_{A_2}$ on the spread of HIV infection respectively. We can clearly note that ${\mathcal {R}}_{0}$ is non-negative as $h_3h_4>\gamma _1\gamma _2$ which implies that $\Phi <1$.

#### Local stability of the disease-free steady state

The following theorem follows from van den Driessche and Watmough [10] (Theorem 2).

##### Theorem 2

*The disease-free equilibrium point*${\mathcal {D}}^f$*of model system equations* (3) *is locally asymptotically stable if*${\mathcal {R}}_0< 1$*and is unstable if*${\mathcal {R}}_0>1$.

#### Endemic equilibrium

The endemic equilibrium denoted by ${\mathcal {D}}^*=\left( S^*,I^*_1,I^*_2,I^*_{A_1},I^*_{A_2}\right)$ satisfies

6 $$\begin{aligned} \left\{ \begin{array}{l} 0=\Lambda -\lambda ^* S^*-\mu S^*, \\ 0=\lambda ^* S^* -h_1 I^*_1,\\ 0=\delta I^*_1-(\mu +\omega _1)I^*_2-H(I^*_2),\\ 0=\sigma _1 I^*_1-(\mu +\gamma _1)I^*_{A1}+\gamma _2I^*_{A2},\\ 0=H(I^*_2)-(\mu +\gamma _2 +\omega _2)I^*_{A2}+\gamma _1 I^*_{A1}. \end{array} \right. \end{aligned}$$

From the first, third, fourth and fifth equation of (6), we have $S^*,\,I^*_1,\,I^*_{A_1},\,I^*_{A_2}$ expressed in terms of $I^*_2$ as follows

7 $$\begin{aligned} \left\{ \begin{array}{l} S^*=\dfrac{\delta \Lambda (I^*_2 r+1)-h_1 I^*_2 \left( h_2+I^*_2 r \left( \mu +\omega _1\right) \right) }{\delta \mu (I^*_2 r+1)},\,\,I^*_1=\dfrac{I^*_2 \left( h_2+I^*_2 r \left( \mu +\omega _1\right) \right) }{\delta +\delta I^*_2 r},\\ I^*_{A1}=\dfrac{I^*_2 \left( \gamma _2 \delta \sigma _2+h_4 \sigma _1 \left( h_2+I^*_2 r \left( \mu +\omega _1\right) \right) \right) }{\delta \left( h_3 h_4-\gamma _1 \gamma _2\right) (I^*_2 r+1)}\,\,\text{ and }\,\,I^*_{A2}=\dfrac{I^*_2 \left( \delta h_3 \sigma _2+\gamma _1 \sigma _1 \left( h_2+I^*_2 r \left( \mu +\omega _1\right) \right) \right) }{\delta \left( h_3 h_4-\gamma _1 \gamma _2\right) (I^*_2 r+1)}. \end{array} \right. \end{aligned}$$

Substituting expressions (7) into the second equation of (6) leads to the following fourth order polynomial equation

8 $$\begin{aligned} I^*_2\left( \xi _3 I^{*3}_2+\xi _2 I^{*2}_2+\xi _1 I^{*}_2+\xi _0\right) =0. \end{aligned}$$

Solving (8) gives $I^*_2=0$ which corresponds to the disease-free equilibrium or

9 $$\begin{aligned} \xi _3 I^{*3}_2+\xi _2 I^{*2}_2+\xi _1 I^{*}_2+\xi _0=0, \end{aligned}$$

where the coefficients $\xi _i$, $0\le i\le 3$ are given in (10).

10 $$\begin{aligned} \left\{ \begin{array}{l} \xi _0=\mu \delta h_1h_2h_3h_4\left( 1-\Phi \right) \left( 1-{\mathcal {R}}_0\right) ,\\ \xi _1=h_1(\beta _3 h_2(\gamma _2 \delta \sigma _2+h_2 h_4 \sigma _1)-\gamma _1(\gamma _2(\beta _1 h_2^2+\delta h_2(\beta _2+\mu r)+\delta \mu r(\mu +\omega _1))\\ \quad -\beta _4 h_2^2 \sigma _1)+h_3(\beta _4 \delta h_2 \sigma _2+h_4(\beta _1 h_2^2+\delta h_2 (\beta _2+\mu r)+\delta \mu r(\mu +\omega _1))))\\ \quad -\delta \Lambda r(\beta _3 \gamma _2 \delta \sigma _2-2 \beta _2 \gamma _1 \gamma _2 \delta +\beta _4 \gamma _1 \mu \sigma _1-\beta _1 \gamma _1 \gamma _2(h_2+\mu +\omega _1)+\sigma _1 \omega _1(\beta _4 \gamma _1+\beta _3 h_4)\\ \quad +\beta _4 \gamma _1 h_2 \sigma _1+h_3(\beta _4 \delta \sigma _2+h_4(2 \beta _2 \delta +\beta _1(h_2+\mu +\omega _1)))+\beta _3 h_4 \sigma _1 (h_2+\mu )),\\ \xi _2=r(h_1(\beta _3(\mu +\omega _1)(\gamma _2 \delta \sigma _2+2 h_2 h_4 \sigma _1)+\gamma _1(2 \beta _4 h_2 \sigma _1(\mu +\omega _1)-\gamma _2(h_2(\beta _2\delta +2 \beta _1(\mu +\omega _1))\\ \quad +\delta (\mu +\omega _1)(\beta _2+\mu r)))+h_3(\beta _4 \delta \sigma _2(\mu +\omega _1)+h_4(h_2(\beta _2 \delta +2\beta _1(\mu +\omega _1))+\delta (\mu +\omega _1)\\ \quad \times (\beta _2+\mu )))) -\delta \Lambda r (\gamma _1(\beta _4 \mu \sigma _1-\beta _2 \gamma _2\delta )-\beta _1 \gamma _1 \gamma _2(\mu +\omega _1)+\sigma _1\omega _1(\beta _4\gamma _1+\beta _3 h_4)\\ \quad + h_3 h_4 (\beta _2\delta +\beta _1(\mu +\omega _1))+\beta _3 h_4\mu \sigma _1)),\\ \xi _3=r^2(\mu +\omega _1)h_1(\beta _2 \delta (\omega _2h_3+\mu (\gamma _1+\gamma _2+\mu ))+\sigma _1(\mu +\omega _1)(\beta _3h_4+\beta _4\gamma _1)\\ \quad +\beta _1(\mu +\omega _1) (\gamma _1 (\mu +\omega _2)+\mu h_4)). \end{array} \right. \end{aligned}$$

We can clearly note that, $\xi _0>0\Leftrightarrow {\mathcal {R}}_0<1$ and $\xi _0<0\Leftrightarrow {\mathcal {R}}_0>1$. We now determine the number of possible positive real zeros of the polynomial (10) using the Descartes Rule of Signs. The possibilities can be presented as shown below. Here, the number of possible positive real zeros is denoted by $i^{*}$.

**Table:** 

$$\xi _3>0$$								
$\xi _{2} > 0$					$\xi _{2} < 0$			
$\xi _{1} > 0$			$\xi _{1} < 0$		$\xi _{1} > 0$		$\xi _{1} < 0$	
$\xi _{0} > 0$		$\xi _{0} < 0$	$\xi _{0} > 0$	$\xi _{0} < 0$	$\xi _{0} > 0$	$\xi _{0} < 0$	$\xi _{0} > 0$	$\xi _{0} < 0$
$i^{*}$	0	1	2	1	2	3	2	1

#### Backward bifurcation

Theorem 4.1 proven in Castillo-Chavez and Song [11] will be useful. We show that system (3) undergoes a backward bifurcation. Let us make the following change of variables:

$S=x_{1},\,I_1=x_2,\,I_2=x_3,\,I_{A1}=x_4,\,I_{A2}=x_5$, so that $\text{ N }=\displaystyle \sum _{n=1}^{5}{x_n}$. We now use the vector notation $X=\left( x_{1},x_{2},x_{3},x_{4},x_{5}\right) ^{T}$. Then, system (3) can be written in the form

$$\dfrac{dX}{dt}=F(t,x(t))=\left( f_{1},f_{2},f_{3},f_{4},f_{5}\right) ^T$$, where

11 $$\begin{aligned} \left\{ \begin{array}{l} \dfrac{dx_1}{dt}=\Lambda -\dfrac{\left( \beta _1x_2 +\beta _2x_3+\beta _3x_4+\beta _4x_5\right) x_1}{N}-\mu x_1=f_1, \\ \dfrac{dx_2}{dt}=\dfrac{\left( \beta _1x_2 +\beta _2x_3+\beta _3x_4+\beta _4x_5\right) x_1}{N} -h_1x_2=f_2,\\ \dfrac{dx_3}{dt}=\delta x_2-(\mu +\omega _1)x_3-\dfrac{\sigma _2 x_3}{1+rx_3}=f_3,\\ \dfrac{dx_4}{dt}=\sigma _1 x_2-h_3 x_4 + \gamma _2x_5=f_4,\\ \dfrac{dx_5}{dt}=\dfrac{\sigma _2 x_3}{1+rx_3}-h_4x_5 + \gamma _1 x_4=f_5. \end{array} \right. \end{aligned}$$

We now define

12 $$\begin{aligned} \beta _{i+1}=\theta _{i}\beta _1,\,\,i=1,2,3 \end{aligned}$$

with $\theta _i=1$ signifying that the chance of acquiring HIV infection upon contact with individuals in class $x_2$ or upon contact with individuals in classes $x_3$, $x_4$ and $x_5$ is the same, $\theta _i \in (0,1)$ signifying a reduced chance of acquiring HIV infection upon contact with individuals in classes $x_3$, $x_4$ and $x_5$ as compared to individuals in class $x_2$, $\theta _i >1$ signifies an increased rate of acquiring HIV infection upon contact with individuals in classes $x_3$, $x_4$ and $x_5$ as compared to individuals in class $x_2$.

Let $\beta _1$ be the bifurcation parameter, ${\mathcal {R}}_0=1$ corresponds to

13 $$\begin{aligned} \beta _1=\beta ^*_1=\frac{h_1 h_2 \left( h_3 h_4-\gamma _1 \gamma _2\right) }{\gamma _2 \delta \theta _2 \sigma _2-\gamma _1 \gamma _2 \delta \theta _1+\gamma _1 h_2 \theta _3 \sigma _1-\gamma _1 \gamma _2 h_2+\delta h_3 \theta _3 \sigma _2+\delta h_3 \theta _1 h_4+h_2 \theta _2 h_4 \sigma _1+h_2 h_3 h_4}. \end{aligned}$$

The Jacobian matrix of model system (3) at ${\mathcal {D}}_f$ when $\beta _1=\beta ^*_1$ is given by

$$\begin{aligned} J^*({\mathcal {D}}_f)=\left( \begin{array}{ccccc} -\mu &{} -\beta ^* _1 &{} -\beta ^* _1 \theta _1 &{} -\beta ^* _1 \theta _2 &{} -\beta ^* _1 \theta _3 \\ 0 &{} \beta ^* _1-h_1 &{} \beta ^* _1 \theta _1 &{} \beta ^* _1 \theta _2 &{} \beta ^* _1 \theta _3 \\ 0 &{} \delta &{} -h_2 &{} 0 &{} 0 \\ 0 &{} \sigma _1 &{} 0 &{} -h_3 &{} \gamma _2 \\ 0 &{} 0 &{} \sigma _2 &{} \gamma _1 &{} -h_4 \\ \end{array} \right) \end{aligned}$$

where $h_1$, $h_2$, $h_3$ and $h_4$ are defined as before.

Model system (11), with $\beta _1=\beta ^*_1$ has a simple eigenvalue, hence the center manifold theory can be used to analyse the dynamics of model system (3) near $\beta _1=\beta ^*_1$. It can be shown that $J^*({\mathcal {D}}^f)$, has a right eigenvector given by $w=(w_1,w_2,w_3,w_4,w_5)^{T}$, where

$$\begin{aligned} w_1= & {} -h_1h_2h_3h_4\left( 1-\Phi \right) ,\,\,w_2=\mu h_2h_3h_4\left( 1-\Phi \right) ,\,\,w_3=\mu \delta h_3h_4\left( 1-\Phi \right) , \\ w_4= & {} \mu \left( \gamma _2 \delta \sigma _2+h_2 h_4 \sigma _1\right) ,\,\,w_5=\mu \left( \gamma _1 h_2 \sigma _1+\delta h_3 \sigma _2\right) . \end{aligned}$$

Here, we note that $w_1<0$ and $w_i>0,\,\,i=2,3,4,5$. Further, the left eigenvector of $J^*({\mathcal {D}}^f)$, associated with the zero eigenvalue at $\beta _1=\beta ^*_1$ is given by $v=(v_1,v_2,v_3,v_4,v_5)^{T}$, where

$$\begin{aligned} v_1=\, & {} 0, \\ v_2= & {} \gamma _2 \delta \left( \theta _2 \sigma _2-\gamma _1 \theta _1\right) +h_2 \left( -\gamma _1 \gamma _2+\sigma _1 \left( \gamma _1 \theta _3+h_4 \theta _2\right) +h_3 h_4\right) +\delta h_3 \left( h_4 \theta _1+\theta _3 \sigma _2\right) ,\\ v_3= \,& {} h_1 \left( \gamma _2 \left( \theta _2 \sigma _2-\gamma _1 \theta _1\right) +h_3 \left( h_4 \theta _1+\theta _3 \sigma _2\right) \right) , \\ v_4=\, & {} h_1 h_2 \left( \gamma _1 \theta _3+h_4 \theta _2\right) ,\,\,v_5=\,h_1 h_2 \left( \gamma _2 \theta _2+h_3 \theta _3\right) . \end{aligned}$$

Here, take note that $v_2>0$, $v_3>0$ accordingly as $\sigma _2\theta _2 >\gamma _1\theta _1$ and $v_2<0$, $v_3<0$ accordingly as $\sigma _2\theta _2 <\gamma _1\theta _1$. Also, $v_4>0$ and $v_5>0$.

The computations of **a** and **b** are necessary in order to apply Theorem 4.1 in Castillo-Chavez and Song [11]. For system (11), the associated non-zero partial derivatives of *F* at the disease-free equilibrium are given in (14).

14 $$\begin{aligned} \frac{\partial ^2 f_1}{\partial x^2_1} & = \frac{2 \beta ^* _1 \mu }{\Lambda },\,\,\,\frac{\partial ^2 f_1}{\partial x_2\partial x_3} = \frac{\partial ^2 f_1}{\partial x_3\partial x_2} = \frac{\beta ^* _1 \theta _1 \mu }{\Lambda }+\frac{\beta ^* _1 \mu }{\Lambda }, \nonumber \\ \frac{\partial ^2 f_1}{\partial x_2\partial x_4} & = \frac{\partial ^2 f_1}{\partial x_4\partial x_2} = \frac{\beta ^* _1 \theta _2 \mu }{\Lambda }+\frac{\beta ^* _1 \mu }{\Lambda },\,\,\,\frac{\partial ^2 f_1}{\partial x_2\partial x_5} = \frac{\partial ^2 f_1}{\partial x_5\partial x_2} = \frac{\beta ^* _1 \theta _3 \mu }{\Lambda }+\frac{\beta ^* _1 \mu }{\Lambda }, \nonumber \\ \frac{\partial ^2 f_1}{\partial x^2_3} & = \frac{2 \beta ^* _1 \theta _1 \mu }{\Lambda },\,\,\,\frac{\partial ^2 f_1}{\partial x_3\partial x_4} = \frac{\partial ^2 f_1}{\partial x_4\partial x_3} = \frac{\beta ^* _1 \theta _1 \mu }{\Lambda }+\frac{\beta ^* _1 \theta _2 \mu }{\Lambda }, \nonumber \\ \frac{\partial ^2 f_1}{\partial x_3\partial x_5} & = \frac{\partial ^2 f_1}{\partial x_5\partial x_3}= \frac{\beta ^* _1 \theta _1 \mu }{\Lambda }+\frac{\beta ^* _1 \theta _3 \mu }{\Lambda },\,\,\,\frac{\partial ^2 f_1}{\partial x^2_4}=\frac{2 \beta ^* _1 \theta _2 \mu }{\Lambda }, \nonumber \\ \frac{\partial ^2 f_1}{\partial x_4\partial x_5}& = \frac{\partial ^2 f_1}{\partial x_5\partial x_4}=\frac{\beta ^* _1 \theta _2 \mu }{\Lambda }+\frac{\beta ^* _1 \theta _3 \mu }{\Lambda },\,\,\,\frac{\partial ^2 f_1}{\partial x^2_5}=\frac{2 \beta ^* _1 \theta _3 \mu }{\Lambda }, \nonumber \\ \frac{\partial ^2 f_2}{\partial x^2_1}& = -\frac{2 \beta ^* _1 \mu }{\Lambda },\,\,\,\frac{\partial ^2 f_2}{\partial x_2\partial x_3}=\frac{\partial ^2 f_2}{\partial x_3\partial x_2}=-\frac{\beta ^* _1 \theta _1 \mu }{\Lambda }-\frac{\beta ^* _1 \mu }{\Lambda },\nonumber \\ \frac{\partial ^2 f_2}{\partial x_2\partial x_4}& = \frac{\partial ^2 f_2}{\partial x_4\partial x_2}=-\frac{\beta ^* _1 \theta _2 \mu }{\Lambda }-\frac{\beta ^* _1 \mu }{\Lambda },\,\,\,\frac{\partial ^2 f_2}{\partial x_2\partial x_5}=\frac{\partial ^2 f_2}{\partial x_5\partial x_2}=-\frac{\beta ^* _1 \theta _3 \mu }{\Lambda }-\frac{\beta ^* _1 \mu }{\Lambda }, \nonumber \\ \frac{\partial ^2 f_2}{\partial x^2_3}& = -\frac{2 \beta ^* _1 \theta _1 \mu }{\Lambda },\,\,\,\frac{\partial ^2 f_2}{\partial x_3\partial x_4}=\frac{\partial ^2 f_2}{\partial x_4\partial x_3}=-\frac{\beta ^* _1 \theta _1 \mu }{\Lambda }-\frac{\beta ^* _1 \theta _2 \mu }{\Lambda }, \nonumber \\ \frac{\partial ^2 f_2}{\partial x_3\partial x_5}& = \frac{\partial ^2 f_2}{\partial x_5\partial x_3}=- \frac{\beta ^* _1 \theta _1 \mu }{\Lambda }-\frac{\beta ^* _1 \theta _3 \mu }{\Lambda },\,\,\,\frac{\partial ^2 f_2}{\partial x^2_4}=-\frac{2 \beta ^* _1 \theta _2 \mu }{\Lambda }, \nonumber \\ \frac{\partial ^2 f_2}{\partial x_4\partial x_5}& = \frac{\partial ^2 f_2}{\partial x_5\partial x_4}=-\frac{\beta ^* _1 \theta _2 \mu }{\Lambda }-\frac{\beta ^* _1 \theta _3 \mu }{\Lambda },\,\,\,\frac{\partial ^2 f_2}{\partial x^2_5}=-\frac{2 \beta ^* _1 \theta _3 \mu }{\Lambda }, \nonumber \\ \frac{\partial ^2 f_3}{\partial x^2_3}& = 2 r \sigma _2,\,\,\,\frac{\partial ^2 f_5}{\partial x^2_3}=-2 r \sigma _2, \nonumber \\ \frac{\partial ^2 f_1}{\partial x_2\partial \beta ^*_1}& = -1,\,\,\,\frac{\partial ^2 f_1}{\partial x_3\partial \beta ^*_1}=-\theta _1\,\,\,,\frac{\partial ^2 f_1}{\partial x_4\partial \beta ^*_1}=-\theta _2,\,\,\,\frac{\partial ^2 f_1}{\partial x_5\partial \beta ^*_1}=-\theta _3, \nonumber \\ \frac{\partial ^2 f_2}{\partial x_2\partial \beta ^*_1}& = 1,\,\,\,\frac{\partial ^2 f_2}{\partial x_3\partial \beta ^*_1}=\theta _1\,\,\,,\frac{\partial ^2 f_2}{\partial x_4\partial \beta ^*_1}=\theta _2,\,\,\,\frac{\partial ^2 f_2}{\partial x_5\partial \beta ^*_1}=\theta _3. \end{aligned}$$

It thus follows that

$$\begin{aligned} {\text{ a }} & = \sum\limits_{{i = 2}}^{5} {v_{2} w_{2} w_{i} \frac{{\partial ^{2} f_{2} }}{{\partial x_{2} \partial x_{i} }}} + \sum\limits_{{i = 2}}^{5} {v_{2} w_{3} w_{i} \frac{{\partial ^{2} f_{2} }}{{\partial x_{3} \partial x_{i} }}} + \sum\limits_{{i = 2}}^{3} {v_{2} w_{4} w_{i} \frac{{\partial ^{2} f_{2} }}{{\partial x_{4} \partial x_{i} }}} \\ & + v_{2} w_{5}^{2} \frac{{\partial ^{2} f_{2} }}{{\partial x_{5}^{2} }} + v_{3} w_{3}^{2} \frac{{\partial ^{2} f_{2} }}{{\partial x_{3}^{2} }} + v_{5} w_{3}^{2} \frac{{\partial ^{2} f_{2} }}{{\partial x_{3}^{2} }} \\ & = \frac{{\beta _{1} \mu v_{2} \left( { - \left( {2\left( {w_{2} + w_{3} + w_{4} } \right) + w_{5} } \right)\left( {\theta _{1} w_{3} + \theta _{2} w_{4} + w_{2} } \right) - \theta _{3} w_{5} \left( {w_{2} + w_{3} + w_{4} + 2w_{5} } \right)} \right)}}{\Lambda } \\ & + \quad 2r\sigma _{2} \left( {v_{3} - v_{5} } \right)w_{3}^{2} \\ & = \Theta _{1} - \Theta _{2} = \Theta _{2} \left( {\Delta - 1} \right){\mkern 1mu} {\mkern 1mu} \left( {\frac{{\Theta _{1} }}{{\Theta _{2} }} = \Delta } \right), \\ \end{aligned}$$

where

$$\begin{aligned} \Theta _1 & = 2 r \sigma _2 v_3w_3^2, \\ \Theta _2= & {} \frac{\beta _1 \mu v_2}{\Lambda } \left( \left( 2 \left( w_2+w_3+w_4\right) +w_5\right) \left( \theta _1 w_3+\theta _2 w_4+w_2\right) +\theta _3 w_5 \left( w_2+w_3+w_4+2 w_5\right) \right) \\&+2 r \sigma _2 v_5w_3^2. \end{aligned}$$

Note that if $\Delta >1$, then $\text{ a }>0$ and if $\Delta <1$ then $\text{ a }<0$. Lastly,

$$\begin{aligned} \text{ b }= & {} \displaystyle \sum ^5_{i=2}{v_2w_i\frac{\partial ^2f_2}{\partial x_i\partial \beta ^*_1}}=\mu (\delta h_3 h_4(\theta _1+\theta _2)(1-\Phi )\\&+\,h_2(\gamma _1 \theta _3 \sigma _1 + h_3 h_4(1-\Phi ))+\delta h_3 \theta _3 \sigma _2)(\delta (\theta _1 (\gamma _1 (\mu +\omega _2)+h_4 \mu ) \\&+\,\sigma _2 (\gamma _2 \theta _2+h_3 \theta _3))+h_2 (h_3 h_4(1-\Phi )+\sigma _1 (\gamma _1 \theta _3+h_4 \theta _2)))>0. \end{aligned}$$

We thus have the following result

##### Theorem 3

*If*$\Delta >1$, *then system* (3) *has a backward bifurcation at*${\mathcal {R}}_0=1$. *Otherwise, if*$\Delta <1$*the endemic equilibrium is locally asymptotically stable for*${\mathcal {R}}_0>1$*but close to one.*

**Fig. 1:**
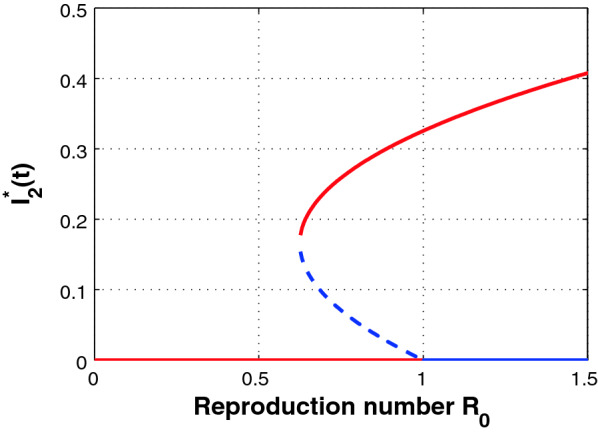
The figure showing a backward bifurcation. The *solid lines* denote stable states and the *dotted lines* denote unstable states

We show the existence of a backward bifurcation through numerical example by creating bifurcation diagram around ${\mathcal {R}}_0 =1$ (Fig. 1). To draw a bifurcation curve (the graph of $I^*_2$ as a function of ${\mathcal {R}}_0$), we fix the following parameters for illustrative purposes: $\Lambda = 0.25,\,\mu = 0.03,\,\beta _1 = 0.5,\,\beta _2 = 0.4,\,\beta _3 = 0.4,\,\beta _4 = 0.2,\,\delta = 0.7,\,\sigma _1 = 0.009,\,\sigma _2 = 0.04,\,r = 0.5,\,\omega _1 = 0.09,\,\omega _2 = 0.06,\,\gamma _1 = 0.009,\,\gamma _2 = 0.09$.

##### Remark

Epidemiologically, when a model exhibits backward bifurcation, this entails that it is not enough to only reduce the basic reproductive number to less than one in order to eliminate the disease.

## Results and discussion

### Numerical simulations

We carry out numerical simulations to support our theoretical findings.

### Estimation of parameters

Parameter values used for numerical simulations are given in Table 1.

**Table 1:** Parameter values used in numerical simulations

Parameter	Definition	Range	Value	Source
$\beta _1$	Contact for individuals in *S* with those in $I_1$	0-1	0.912	[8]
$\beta _2$	Contact for individuals in *S* with those in $I_2$	0-1	0.894	[8]
$\beta _3$	Contact for individuals in *S* with those in $I_{A1}$	$0{-}1$	0.095	[8]
$\beta _4$	Contact for individuals in *S* with those in $I_{A2}$	$0{-}1$	0.091	[8]
$\sigma _1$	Progression from $I_1$ to $I_{A1}$	$0.01{-}1$	0.084	[8]
$\sigma _2$	Progression from $I_2$ to $I_{A2}$	$0{-}1$	0.1	Assumed
$\delta$	Progression from $I_1$ to $I_{2}$	$0.01{-}1$	1.0	[8]
$\gamma _1$	Progression from $I_{A1}$ to $I_{A2}$	$0.01{-}1$	0.096	[8]
$\gamma _2$	Progression from $I_{A2}$ to $I_{A1}$	$0.1{-}1$	0.112	[8]
*r*	Effect of late initiation into ART	$0{-}1$	0.45	Assumed
$\omega _1$	Disease related death of individuals in $I_2$	$0{-}1$	0.089	[7]
$\omega _2$	Disease related death of individuals in $I_{A2}$	$0{-}1$	0.095	[7]
$\Lambda$	Recruitment rate into *S*	$0{-}1$	0.0239	[12–14]
$\mu$	Natural death rate	$0{-}1$	0.0172	[14]

### Numerical results

**Fig. 2:**
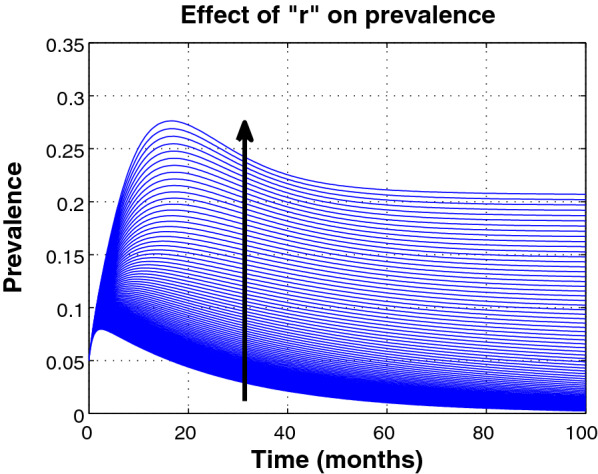
Effect of varying parameter *r* on the prevalence of HIV, starting from 0.1 up to 1.0 with a step size of 0.01

Figure 2 illustrates the effect of varying the parameter *r* on the prevalence of HIV. We note that increasing the parameter *r* results in an increase in the prevalence of HIV. In particular, increasing *r* from 0.1 up to 1.0 increases the prevalence rate of HIV with a level of approximately $28\%$. This is a reflection that late diagnosis of HIV contributes to an increase in HIV infections. Thus, more effort should be directed towards encouraging individuals to get tested for HIV and ensuring those who are positive are timely initiated into ART treatment.

## Conclusions

A mathematical model that describes the dynamics of HIV/AIDS has been formulated using nonlinear ordinary differential equations. The model takes into account the impact of late diagnosis on HIV/AIDS transmission dynamics. Initiation into ART treatment of individuals with a CD4^+^ T cell count in the range 200–350\μ L has been described by the function (1). The model developed in this paper fits well with settings in most underdeveloped countries where stigma of HIV remains prevalent. Inclusion of the treatment function (1) increases the realism of the model developed by [8] and leads to some interesting dynamical aspects such as the occurrence of backward bifurcation.

In this study, it has been shown that the classical ${\mathcal {R}}_0$—threshold is not the key to control the spread of HIV infection within a population. In fact HIV infection may persist in the population even with subthreshold values of ${\mathcal {R}}_0$. Our results suggest that considerable effort should be directed towards encouraging early initiation into ART in order to reduce HIV prevalence. For instance, strategies such as the implementation of HIV self-testing programs would be of great help in the fight against HIV.

## Limitations

Like in any model development, the model is not without limitations. The model can be extended to include the contribution of pre-exposure prophylaxis (PrEP) and other control measures not considered in the work.

## Data Availability

Estimation of parameters have been stated throughout the body of the paper and included in the reference section. The graphs were produced using the MATLAB software that is available from https://www.mathworks.com/products/matlab.html.

## Abbreviations

AIDS: Acquired immune deficiency syndrome
HIV: Human immunodeficiency virus
ART: Antiretroviral therapy
